# Intercalary staphyloma after strabismus surgery in a patient with Marfan syndrome: A case report

**DOI:** 10.1097/MD.0000000000029468

**Published:** 2022-06-17

**Authors:** Ju Mi Kim, Jae-young Kim, Jae-yeon Sung, Jae Yul Hwang, Yeon-Hee Lee

**Affiliations:** a Department of Ophthalmology and Visual Science, College of Medicine, Daejeon St. Mary's Hospital, The Catholic University of Korea, Seoul, Korea; b Department of Ophthalmology, Chungnam National University Hospital, Korea; c Department of Ophthalmology, Chungnam National University Sejong Hospital, Korea.

**Keywords:** case report, intercalary staphyloma, Marfan syndrome, strabismus

## Abstract

**Rationale:**

A few cases of intercalary staphyloma have been reported in patients with Marfan syndrome, but we believe that this is the first case of intercalary staphyloma in Marfan syndrome developing after strabismus surgery.

**Patient concerns:**

A 9-year-old girl diagnosed with Marfan syndrome visited a strabismus clinic for treatment of esotropia. Both eyes were aphakic and had 60 prism diopter esotropia at distance and 55 prism diopter esotropia at near. There were no corneal, conjunctival, or scleral abnormalities. Six millimeters of recession was performed on both medial rectus muscles via an inferonasal fornix approach under general anesthesia. 5 days after surgery, a dark gray protruding lesion was observed on the upper nasal side of the left eye.

**Diagnoses:**

Intraocular ultrasonography showed no bleeding, retinal detachment, or other abnormal findings. Computed tomography showed a conical protrusion of the scleral wall which was diagnosed as intercalary staphyloma.

**Interventions:**

To reduce risk of progression of the staphyloma in the left eye and to reduce risk of development of a new staphyloma, intraocular pressure lowering eye drops were administered.

**Outcomes:**

We just observed it without any intervention except the intraocular pressures lowering eye drops. It remained stable for 12 months.

**Lessons:**

Clinicians need to be alert to the possibility of this serious complication in Marfan syndrome patients after minor surgical trauma, which can occur during uneventful strabismus surgery.

## 1 Introduction

The ocular abnormalities of Marfan syndrome are very common and characteristic; almost 50% of patients with Marfan syndrome are diagnosed primarily during evaluations for ophthalmic complaints.^[1]^ Typical ophthalmic abnormalities include lens deviation, glaucoma, retinal detachment, myopia, corneal astigmatism, a thin sclera, and strabismus.^[2]^ Strabismus occurs in 19–45% of patients with Marfan syndrome, compared with 3–5% of normal controls.^[3,4]^ Therefore, patients with Marfan syndrome often require strabismus surgery, and it is necessary that clinicians are aware of the complications associated with this surgery. We experienced a case of intercalary staphyloma that developed after strabismus surgery in a patient with Marfan syndrome and share our experience herein.

## 2 Case report

**Figure 1:**
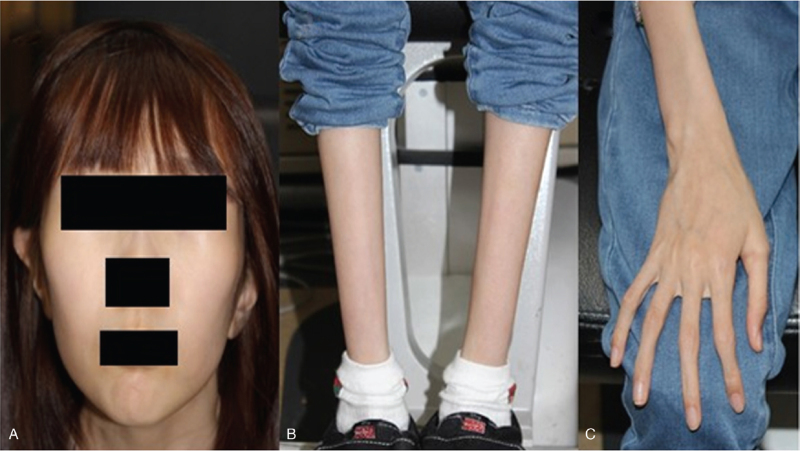
The classical signs of Marfan syndrome in our patient included (A) dolichocephaly (long, thin skull), (B) dolichostenomelia (long, thin extremities), and (C) arachnodactyly (spiderlike digits).

A 9-year-old girl came to our clinic for treatment of esotropia. She was diagnosed with Marfan syndrome with a confirmed *FBN1* gene mutation (Fig. 1). Mitral and tricuspid regurgitation was observed along with aortic root ectasia, and she had undergone mitral valve replacement 2 years previously. She also wore a brace for scoliosis.

Her crusting, scaling, and erythematous lid margins suggesting that she had chronic blepharitis. Both eyes were aphakic, with a refractive error of +9D in the right eye and +8D in the left. Her corrected visual acuity was 12/20 in the right eye and 8/20 in the left; the intraocular pressures were normal at 15 mm Hg in the right eye and 16 mm Hg in the left eye.

**Figure 2:**
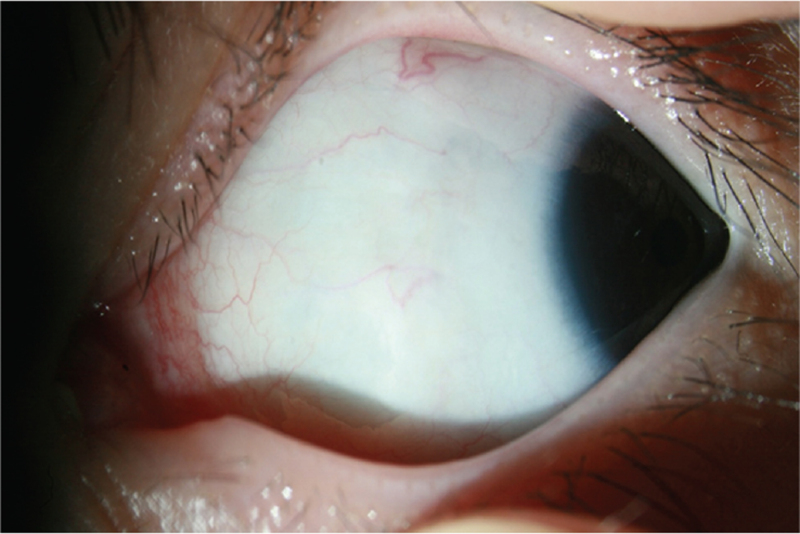
Slit lamp photograph of the patient's left eye at the first visit. There were no abnormal findings in the cornea, conjunctiva, or sclera.

Neither pupil dilated well. The maximum pupil diameter was 3 mm after instilling a mydriatic. The axial length was rather long, which was 28.76 mm for the right eye and 30.34 mm for the left eye. Areas of retinal pigment epithelium depigmentation were seen in both fundi. There were no abnormalities in the cornea, conjunctiva, or sclera, including a blue sclera (Fig. 2). She had 60 prism diopter (PD) esotropia at distance and 55 PD esotropia nearby. The deviation angle was 45 PD esotropia at distance after correcting the refractive error with glasses. Her parents wanted surgical esotropia correction to be performed to improve her appearance.

Six millimeters of recession was performed on both medial rectus muscles via an inferonasal fornix approach under general anesthesia. There was no marked thinning of the sclera, and the conventional surgery was performed gently and was uneventful. On the day after surgery, there were no abnormalities in the cornea, conjunctiva, sclera, or anterior chamber, except hyperemia and minor hemorrhage around the surgical wound.

**Figure 3:**
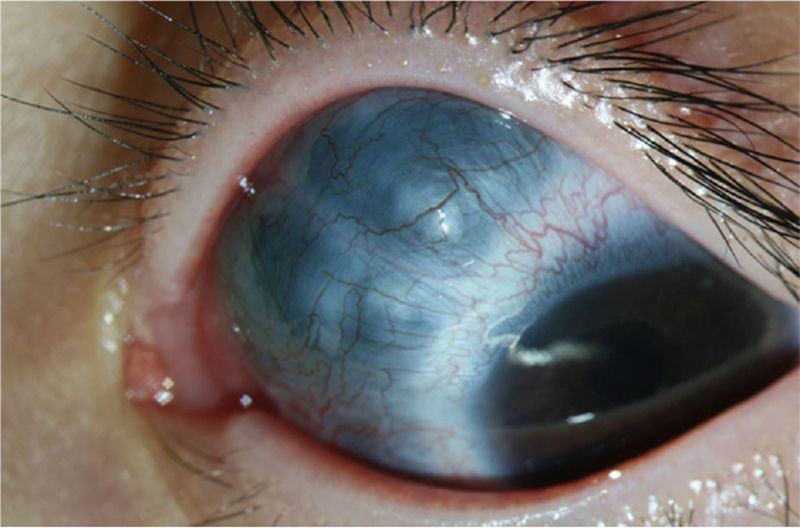
Slit lamp photograph showing the intercalary staphyloma. A dark gray protruding lesion was observed on the superonasal side of the left eye, from 7 to 11 o’clock.

**Figure 4:**
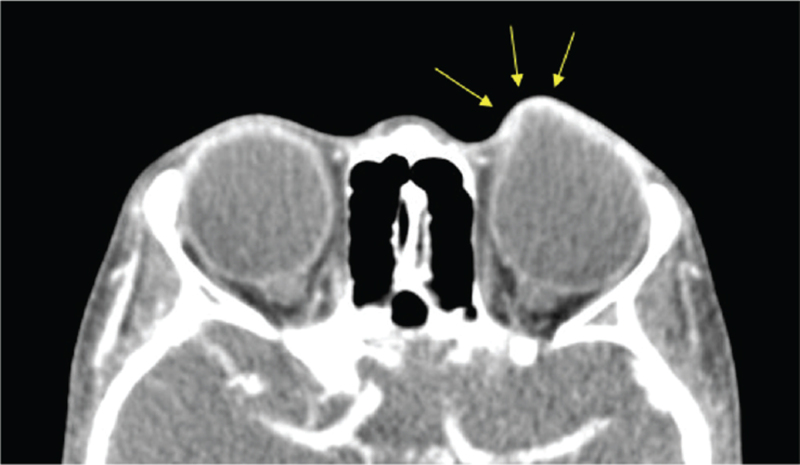
Facial computed tomography showing conical protrusion of the intercalary staphyloma (arrow).

Five days after surgery, a dark gray protruding lesion was observed on the upper nasal side of the left eye (Fig. 3), from 7 to 11 o’clock. On slit lamp microscopy, dark gray uveal tissue was seen though the thinned sclera and conjunctiva. The surface was uneven with several small protrusions. Intraocular ultrasonography showed no bleeding, retinal detachment, or other abnormal findings. Computed tomography showed a conical protrusion of the scleral wall (Fig. 4), which was diagnosed as intercalary staphyloma.

The intraocular pressure on the 5th day postoperatively was 21 mm Hg in the right eye and 22 mm Hg in the left eye, and the intraocular pressures were maintained at an upper limit of normal range or slightly higher than normal. To reduce risk of progression of the staphyloma in the left eye and to reduce risk of development of a new staphyloma, intraocular pressure lowering eye drops were administered. We just observed it without any intervention except the intraocular pressures lowering eye drops. It remained stable for 12 months.

## 3 Discussion

Marfan syndrome is an autosomal dominant disease characterized by musculoskeletal and ocular abnormalities, and cardiovascular disease.^[1]^ It is a disorder of the connective tissue proteins fibrillin-1 and transforming growth factor-β that leads to abnormal connective tissue in many organs.^[5]^ Fibrillin is widely distributed in ocular connective tissue, as reflected by the myriad ocular abnormalities found in Marfan syndrome.^[6]^ The axial myopia and often appeared bluish sclera in Marfan syndrome are postulated to occur due to the weakened, thinned sclera.^[7,8]^

Staphylomas may occur as isolated congenital conditions, or in association with retinal detachment, myopia, glaucoma, scleritis, scleromalacia perforans, trauma, or surgery.^[9]^ The development of a staphyloma is potentiated where the sclera is weakened by the passage of blood vessels.^[9]^

An intercalary staphyloma is a staphyloma that develops between the ciliary body and limbus, where the sclera is weakened by the presence of the anterior ciliary veins and Schlemm's canal.^[9,10]^ Because the sclera is relatively vulnerable in Marfan syndrome, it is not unusual for an intercalary staphyloma to occur after surgery or trauma, since the intercalary region of the sclera is a weaker anatomic site.^[12]^ However, only a few cases of intercalary staphyloma have been reported in Marfan syndrome.^[11–13]^ Goldberg and Ryan^[12]^ first reported a patient with Marfan syndrome and progressive intercalary staphyloma, which was probably related to previous blunt trauma. A 5-year-old white girl with previously diagnosed Marfan syndrome had an intercalary staphyloma. The intercalary staphyloma was on superior part of her right eye extending clockwise from the 9- to 4:30- o’clock meridians. Her right eye had been struck by a stone two months previously. She was seen by an ophthalmologist immediately after the trauma, and no staphyloma, limitation of motion, or proptosis had been observed. Seelenfreund et al^[11]^ and Sahay et al^[13]^ reported the development of intercalary staphylomas following lens extraction surgery in Marfan syndrome patients. In the report by Seelenfreud et al,^[11]^ a 9-year-old girl underwent binocular surgery and, 3 years later, a staphyloma developed on the superior side of her left eye. Sahay et al^[13]^ reported that a 28-year-old patient with Marfan syndrome underwent intracapsular cataract extraction and, 3 years later, intercalary staphyloma extending from 11 to 2 o’ clock was found on his right eye.

Our case is distinct from the previously reported cases in several ways. First, to our knowledge, this is the first report of an intercalary staphyloma in a patient with Marfan syndrome that occurred after strabismus surgery. Second, the previous cases have definite history of scleral traumas that are presumed to be direct causes of the stapylomas. The present case had a strabismus surgery. It was uneventful and performed gently. We believe that the scleral trauma related to the surgery was minor compared to the previously reported cases. Third, the staphyloma developed very abruptly. It developed within a week. It is far faster than previous reports.

At present, it is difficult to fully explain the extremely rapid progression theoretically or empirically. However, it might be caused by underlying weakness of sclera containing abnormal fibrillin molecules. In particular, since the axial length of the patient's right and left eye was 28.76 and 30.34 mm respectively before the strabismus surgery, it is presumed that the sclera was already thinner than normal sclera. In addition, her intraocular pressure was maintained at an upper limit of normal range or slightly higher than normal, so that the patient's thin and weak sclera might have been intolerable. Previous report showed that matrix metalloproteinases might also contribute to degradation of the products of the defective fibrillin-1 gene as compared with normal fibrillin.^[14]^ The patient has suffered from chronic blepharitis and presented with crusting, scaling, and erythematous lid margins. It has been reported that matrix metalloproteinases are highly expressed in the ocular surface of patients with blepharitis.^[15]^

Here, we report a case of intercalary staphyloma in a Marfan syndrome patient that developed after strabismus surgery. Clinicians need to be alert to the occurrence of this serious complication in Marfan syndrome patients after minor surgical trauma, which can occur during uneventful strabismus surgery.

## Author contributions

**Conceptualization:** Yeon-Hee Lee.

**Data curation:** Ju Mi Kim, Jae-young Kim, Yeon-Hee Lee.

**Formal analysis:** Jae-yeon Sung, Jae Yul Hwang

**Investigation:** Ju Mi Kim, Jae-young Kim, Yeon-Hee Lee.

**Methodology:** Yeon-Hee Lee.

**Supervision:** Yeon-Hee Lee.
